# Training in laparoscopic colorectal surgery: a new educational model using specially embalmed human anatomical specimen

**DOI:** 10.1007/s00464-012-2158-y

**Published:** 2012-01-28

**Authors:** Juliette C. Slieker, Hilco P. Theeuwes, Göran L. van Rooijen, Johan F. Lange, Gert-Jan Kleinrensink

**Affiliations:** 1 Department of Surgery, Erasmus University Medical Center, Rotterdam, The Netherlands; 2 Department of Anatomy and Neurosciences, Erasmus University Medical Center, Rotterdam, The Netherlands; 3 Erasmus MC, Laboratory of Experimental Surgery, Room Ee 173, Postbus 2040, 3000 CA Rotterdam, The Netherlands

**Keywords:** Abdominal, Laparoscopic education, Training, Colorectal surgery, Gastrointestinal

## Abstract

**Background:**

With an increasing percentage of colorectal resections performed laparoscopically nowadays, there is more emphasis on training “before the job” on operative skills, including the comprehension of specific laparoscopic surgical anatomy. As integration of technical skills with correct interpretation of the anatomical image must be incorporated in laparoscopic training, a human specimen training model with special emphasis on surgical anatomy was developed.

**Methods:**

The new embalming method Anubifix^™^ combines long-term high-quality embalming of human bodies with almost normal flexibility and plasticity, and the body can be kept operational as long as conventionally embalmed human specimens. A colorectal training model was created in a specimen in which anatomical landmarks of colorectal anatomy were permanently colored to explore laparoscopic colorectal anatomy in a skills training setting. Airtight closure of the abdominal wall permits the creation of pneumoperitoneum. Residents were asked to test the model by mobilizing the small and large bowels and expose the central vessels and ureters. Afterward they were asked to fill out an eight-item questionnaire about the model.

**Results:**

Eleven surgical residents in their first and second year of training participated. Responses to the questionnaire showed that a majority of residents considered the model to be representative of the real situation and superior to animal models or virtual reality simulators, and helped to improve the knowledge of three-dimensional anatomy and laparoscopic skills.

**Conclusion:**

The new training model for laparoscopic colorectal surgery proved to be a high-quality tool, concentrating on laparoscopic colorectal anatomy in a skills training setting. We believe it may be a valuable adjunct to residency training programs based on the principle of “training before the job.”

An increasing percentage of colorectal resections are performed laparoscopically nowadays [1, 2]. Therefore, there is more emphasis on achieving the necessary laparoscopic skills during residency, reducing iatrogenic complications. Recognition of complex surgical anatomy, specific cognitive and motor skills, hand–eye coordination, adjustment to the loss of a degree of tactile feedback, and image interpretation are the principal skills to be acquired by the novice laparoscopic surgeon. Many different training models for laparoscopy have been studied, ranging from box and virtual reality simulators, animal models, and human bodies donated for science. These different training models are all individually useful in training specific aspects of laparoscopy. Box trainers or virtual reality simulators are well suited for training technical skills and hand–eye coordination but lack the training with tissue handling, surgical field interpretation, and anatomical recognition. Animal models are superior for the latter but have strong ethical and financial restrictions and the anatomical situation differs. The abdominal wall of conventionally an embalmed human anatomical specimen is too rigid to create a pneumoperitoneum, and a fresh-frozen specimen or a specimen embalmed via the Thiel method cannot be kept operational for a long period, making them less suitable for training (fresh-frozen lasts 1–2 days and Thiel lasts 3–6 months).

In addition to training surgical skills, training with respect to surgical anatomy has also been emphasized in the past few years. Problem-based surgical anatomy courses have been developed for residents [3] in the Netherlands, even resulting in a national institute for the development and teaching surgical anatomy: the Lowlands Institute for Surgical Anatomy (LISA).

During colorectal laparoscopy, combining cognitive and motor skills with anatomical recognition is challenging for the resident but nonetheless essential for a successful procedure. We aimed to develop a training model for laparoscopic colorectal surgery using human bodies donated for science that were treated with a new embalming method that keeps tissues flexible and soft. In such model, education on laparoscopic cognitive and motor skills can be combined with laparoscopic surgical anatomy.

## Methods

### Model

An anatomic specimen embalmed by means of the Anubifix^™^ method was used. This embalming technique is based on a new prerinsing method combined with a normal 4% formaldehyde fixation solution. In contrast to conventional embalming methods, Anubifix^™^ embalming results in a very small decrease in flexibility and plasticity. Furthermore, this result is accomplished without impairing the quality and duration of conservation. Anubifix^™^ embalming results in a preserved range of motion of joints and flexibility of the abdominal wall combined with tissue tactility comparable to fresh-frozen tissues, this all in contrast to conventional embalming methods.

After the embalming phase, a midline laparotomy of 20 cm was performed. The specific aspects of the colorectal anatomy were dissected and marked [aorta and iliac arteries (dark red), superior mesenteric artery/vein and its branches (red/blue), inferior mesenteric artery/vein and its branches (red/blue), gonadal arteries (purple), and ureters (yellow)]. Coloring of the vessels and ureters was performed circumferentially with a specially developed formaldehyde-proof paint (FPP^™^). After dissection and coloring, the abdominal muscle wall was separated from the overlying fat and skin and closed with running sutures. A rectangular sheet of synthetic butyl rubber measuring 26 × 6 cm with a circular hole at the level of the umbilicus was sutured on top of the sutured muscle wall, analogous to an onlay mesh for incisional hernia, after which the skin was closed with running sutures. The use of a butyl rubber sheet results in an airtight closure of the abdominal wall permitting the creation of a pneumoperitoneum despite the prior abdominal opening. A 10-mm trocar was placed at the umbilicus through which a 30º scope was placed. A standard set of laparoscopic instruments was used. Four 5-mm trocars were placed in the right and left upper quadrants. Pneumoperitoneum was achieved with a continuous flow of CO_2_, with the pressure set between 12 and 15 mmHg, i.e., comparable to the in vivo situation.

### Training course

Surgical residents taking the mandatory national LISA course module Surgical Anatomy of the Colon and Rectum were asked to participate. The LISA course presented here was given in the SkillsLab of the Erasmus University Medical Center, Rotterdam, a clinical training center that includes six modern equipped laparoscopic working stations, a microsurgical lab, and three fully equipped dissection rooms. The LISA course on Surgical Anatomy of the Colon and Rectum is given twice a year to residents in their second or third year of training in general surgery to enhance their knowledge of anatomy, aiming for applied surgical anatomy education. Teaching is always provided by an anatomist and a surgeon.

In the present study, each resident alternated in the role of operating surgeon and first assistant using the model as described above. They were asked to mobilize the small bowel to the left or to the right depending on what side of the colorectal anatomy was to be exposed. Next, the left- and right-sided anatomy was explored, locating segmental colonic anatomy and exposing central vessels and ureters, helped by the coloring and supervised by a faculty member (surgeon as well as anatomist).

### Outcome measures

Following the course, a standardized, anonymous questionnaire was given to analyze the relevance of the course by means of eight questions listed in Table 1. Data were collected using the Likert scale (1 = strongly disagree, 2 = disagree, 3 = neither agree nor disagree, 4 = agree, 5 = strongly agree). Data are presented as mean ± standard deviation.

**Table 1:** Questionnaire following the course (Likert scale 0–5)

The tissue quality is representative for the real situation
The color quality is representative for the real situation
The operative tactility is representative for the real situation
This model helps me improve my knowledge of anatomy for laparoscopic colorectal resection
This model helps me improve my laparoscopic skills
This laparoscopic model is superior to an animal model
This laparoscopic model is superior to virtual reality simulators
The course on laparoscopic surgical anatomy was useful
I would like to see this laparoscopic model implemented in the LISA surgical anatomy course

## Results

**Fig. 1:**
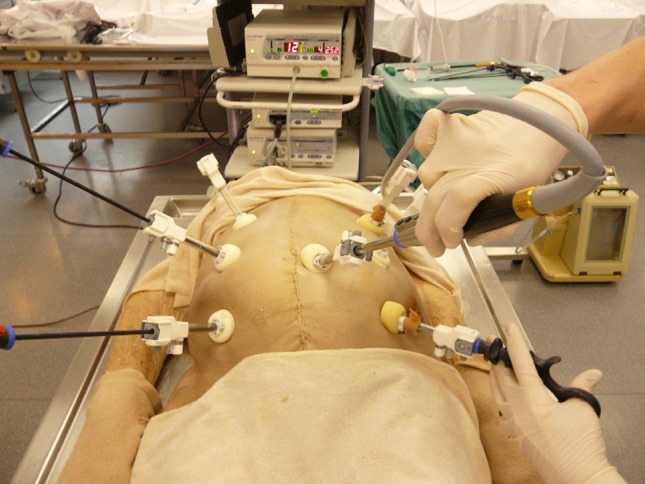
Anubifix™ model laparoscopic colorectal surgery

**Fig. 2:**
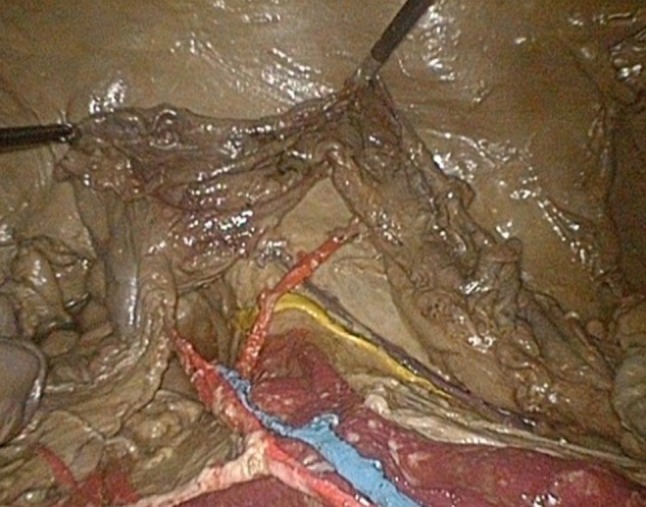
Cecum with ileocolic artery (*red*), ileocolic vein (*blue*), right ureter (*yellow*), and right gonadal artery (*purple*)

**Fig. 3:**
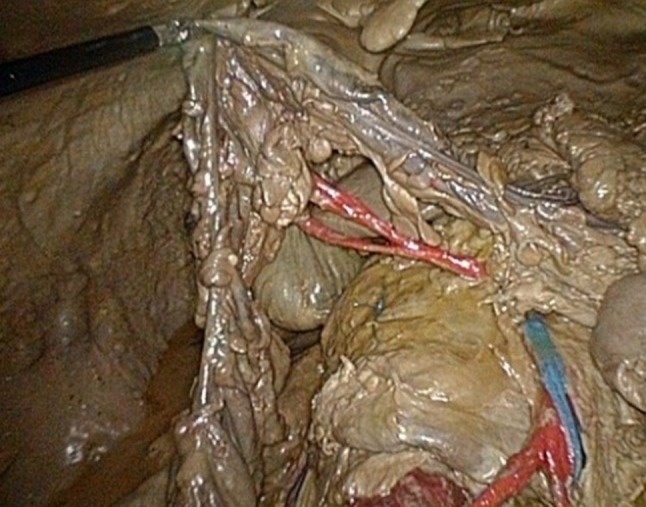
Hepatic flexure with medial colic artery (*red*) and superior mesenteric vein (*blue*)

**Fig. 4:**
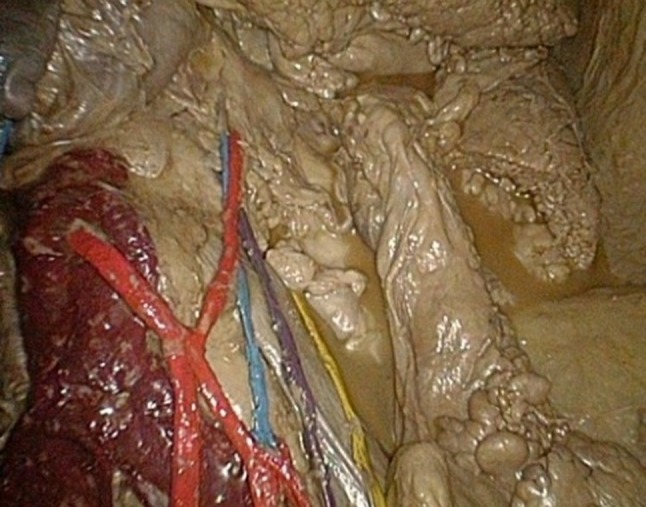
Aorta (*dark red*), inferior mesenteric artery with ascending and descending branch (*red*), inferior mesenteric vein (*blue*), left ureter (*yellow*), and left gonadal artery (*purple*)

**Fig. 5:**
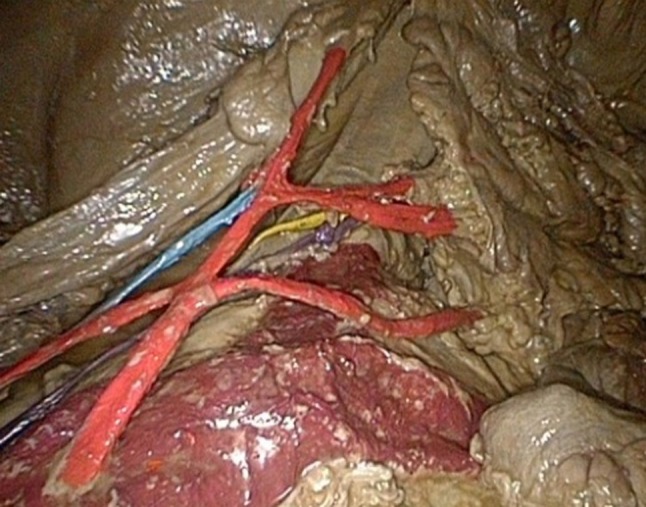
Aorta and iliac arteries (*dark red*), inferior mesenteric artery with descending branch, sigmoid arteries, and superior rectal artery (*red*), inferior mesenteric vein (*blue*), left ureter (*yellow*), and left gonadal artery (*purple*)

The model is shown in Figs. 1, 2, 3, 4 and 5: exterior (Fig. 1), the right-sided anatomy (Figs. 2, 3), and the left-sided anatomy (Figs. 4, 5).

Participants were asked to answer eight questions (Table 1) regarding their perceptions of this model for training colorectal laparoscopy. The questions focused on their opinion of the quality of the tissue and the operative tactility, the gains in knowledge of anatomy and in laparoscopic skills, the comparison to animal models and virtual reality models, and on the utility of implementing this model in a course for surgical anatomy. Eleven surgical residents participated and filled in the questionnaire. Residents had not yet performed laparoscopic colectomies themselves; however, all residents had box training and virtual reality experience.

**Fig. 6:**
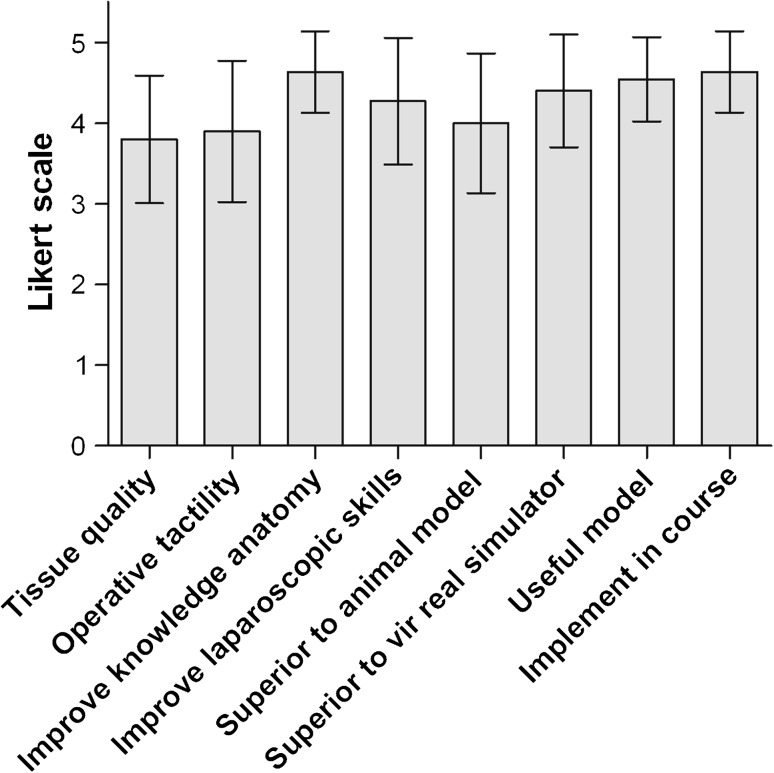
Results of the questionnaire of participants having tested the Anubifix™ model on laparoscopic colorectal surgery (mean ± standard deviation)

The results of the questionnaire, given in Fig. 6, show that the majority of residents believed that (1) the quality of the tissue is representative of the real situation, (2) the model is superior to animal models or virtual reality simulators, and (3) the model helps improve knowledge of three-dimensional anatomy and laparoscopic skills.

## Discussion

The initial concerns about laparoscopic colorectal surgery regarding safety, degree of oncologic resection, and tumor recurrence are shown to be unfounded [4–6], and nowadays an increasing percentage of all colorectal resections is performed through laparoscopic surgery [1, 2]. Laparoscopic colorectal resection has become a successful common surgical procedure as it has many advantages over conventional surgery, including less postoperative pain, earlier recovery of bowel function, less blood loss, and shorter hospital stay [5, 7–9]. However, it remains a technically challenging procedure, and a greater prominent role has been ascribed to training “before the job.” The recognition of the relatively long and technically demanding learning curve has led to, for example, the NICE guidelines which recommend that laparoscopic surgery should be performed only by surgeons who have completed appropriate training and who perform this procedure often enough to maintain competence [10]. In a study performed by Hwang et al. [11], difficulties in laparoscopic performance were evident during the early period of training; an average of ~20 s and two to three errors were required for one grasp. Such results emphasize the need for training residents in a safe tension- and blame-free environment outside the operating room.

Currently, different devices and models exist for training purposes: (1) box trainers and virtual reality simulators, (2) animal models, and (3) models using embalmed anatomical specimens. Box trainers and virtual reality simulators are particularly useful for learning basic optical and manipulative skills. Studies have shown that simulation-based training can be successful in achieving transfer of skills from the training institute to the operative setting [12]. However, not all studies have found a universal improvement for the trained groups; some outcome measures were found to be unchanged after training with a box trainer or virtual reality simulator. A study by Kimura et al. [13] showed that training with a virtual reality simulator or a box trainer was not immediately helpful for shortening the operating time of a laparoscopic cholecystectomy; another study indicated that simulator training did not provide skills for the operating room [14]. A possible explanation for this is that although box trainers or virtual reality simulators focus largely on technical skills, the acquisition of technical skills is only one aspect of laparoscopic training. Laparoscopic surgery not only requires optical and manipulative skills, but also additional cognitive and motor skills with the requirement of long periods of continuous concentration, anatomical image interpretation, and adjustment to the loss of a degree of tactile feedback and altered anatomical view [15]. These difficult laparoscopic requirements need to be practiced and automatic in order to perform a successful procedure.

Animal models offer the possibility to combine technical skills with image interpretation and adjustment to the loss of a degree of tactile feedback under physiologic conditions. However, animal models have important disadvantages: different anatomy, high costs, and in some countries there is active ethical opposition combined with prohibitive legislation against animal models.

Models using embalmed anatomic specimens have the advantage of combining human anatomy, including the experience of normal dimensions, (almost) normal tissue handling, and the experience of normal instruments of choice. The absence of active bleeding can be an issue, although the importance of active bleeding in a training model can be questioned. Until now the most important disadvantage of anatomic specimen models was the conventional embalming method resulting in stiffness and rigidity, hence making it impossible to create a pneumoperitoneum necessary to create an appropriate working space. This problem can be solved by using fresh cadavers or Thiel-embalmed cadavers, but those can be used for only a short period of time (1 day or 3–6 months, respectively). The new embalming method Anubifix^™^, invented in the Anatomy Department of the Erasmus University Medical Center, offers a series of possibilities as it combines long-term high-quality embalming with normal flexibility and plasticity. Because an Anubifix^™^ specimen can be kept operational as long as conventional specimens, unique training models can be made that can be used for many years. In this way the time invested in making such models is returned in having a useful model for training surgical procedures for years, particularly in comparison to Thiel-embalmed human bodies.

Anatomy courses during residency are becoming more important, as it is crucial for young residents to have an excellent knowledge of 3D anatomy of the surgical field. Therefore, short courses in the dissection room during the first years of residency are highly valuable to making teaching in the operating room more efficient. Since a large part of interventions are performed by laparoscopy nowadays, we realized that teaching open anatomy in a human anatomical specimen is clearly no longer sufficient. This is the primary goal of this model: integration of technical skills with correct interpretation of the anatomical image This is lacking in box trainers or virtual reality simulators, while recognition of anatomical landmarks is difficult but essential in laparoscopy. In the training model described here, anatomical landmarks were permanently colored with a specially developed formaldehyde-proof paint to help the resident explore laparoscopic colorectal anatomy in a skills-training setting. We asked 11 residents in their first years of residency to test the model by exploring anatomy, defining segmental colonic anatomy, and exposing the central vessels and ureters. Results show that the majority of the residents found the quality of the tissue representative of the real situation, the model was superior to animal models of virtual reality simulators, and the model was useful for improving knowledge of anatomy and laparoscopic skills.

Chang et al. [16] showed that the majority of residents do not choose to practice in a training lab, although their hospital offers such facility, due to lack of time. We chose to incorporate this model in the Lowlands Institute for Surgical Anatomy (LISA) course. LISA is a Dutch national institute concentrating on surgical anatomy courses for residents. In this institute, all eight Dutch University Medical Centers cooperate in postgraduate training and education in surgical anatomy. In 2004 the Dutch Association of Surgery (NVvH), made the LISA courses obligatory for all surgical residents in the Netherlands. The LISA course is given several times a year and is related to the different phases of training in general surgery to enhance their knowledge of anatomy, aiming for applied surgical anatomy education. The involvement of surgeon and anatomist is considered an essential element of its success.

Traditionally, surgical skills training took place in the operating room; nowadays a complex combination of laparoscopic cognitive and motor skills, altered anatomical image interpretation, and adjustment to the loss of a degree of tactile feedback calls for training models. Until now the importance of training of laparoscopic anatomical skills “before the job” has always been underestimated and this competence had to be acquired on the patient, which is undesirable and responsible for exaggerated long learning curves. We have presented a new training method developed using an embalmed human specimen through which is it possible to practice laparoscopic skills as well as laparoscopic surgical anatomy for laparoscopic colorectal surgery. We propose a standardized integration of laparoscopic Anubifix^™^-embalmed human models in the mandatory surgical anatomy courses in the Netherlands.
